# *Streptococcus pneumoniae *Serotype 3 among Costa Rican Children with Otitis Media: clinical, epidemiological characteristics and antimicrobial resistance patterns

**DOI:** 10.1186/1471-2431-9-52

**Published:** 2009-08-14

**Authors:** Arturo Abdelnour, Carolina Soley, Silvia Guevara, Nurith Porat, Ron Dagan, Adriano Arguedas

**Affiliations:** 1 Instituto de Atención Pediátrica, San José, Costa Rica; 2 Universidad de Ciencias Médicas, San José, Costa Rica; 3 Pediatric Infectious Disease Unit, Soroka University Medical Center, Beer-Sheva, Israel; 4 Faculty of Health Sciences, Ben-Gurion University of the Negev, Beer-Sheva, Israel

## Abstract

**Background:**

After the introduction of the seven valent-pneumococcal conjugated vaccine into our National Immunization Program, it is important to establish and track local serotype distribution in order to evaluate its impact specially because serotype replacement phenomena has been described.

To describe the clinical, epidemiological and antimicrobial resistance patterns of Costa Rican children with otitis media caused by *Streptococcus pneumoniae *serotype 3.

**Methods:**

Middle ear fluid samples were obtained from Costa Rican children with otitis media who participated in various antimicrobial clinical trials between 1992 and 2007. *Streptococcus pneumoniae *was identified according to laboratory standard procedures. Strains were serotyped and antimicrobial susceptibility to penicillin, amoxicillin, cefuroxime, ceftriaxone, azithromycin and levofloxacin was determined by E-test.

**Results:**

Throughout 1992–2007 a total of 1919 tympanocentesis were performed in children with otitis media (median age: 19 months) and yielded a total of 1208 middle ear isolates. The most common pathogens were: *Streptococcus pneumoniae*, 511 isolates (49%); Non-Typable *Haemophilus influenzae*, 386 isolates (37%); *Moraxella catarrahalis*, 100 isolates (9.5%); and *Streptococcus pyogenes*, 54 isolates (5%). *Streptococcus pneumoniae *serotyping was performed in 346/511 isolates (68%) recovered during years 1999–2006. The most common serotypes were 19F (101/30.0%), 14 (46/13.7%), 3 (34/10.1%), 6B (30/8.9%) and 23F (23/6.8%). Analysis performed per years showed a higher prevalence of serotype 3 *Streptococcus pneumoniae *during the study period 2004 and 2005. During the entire study period (1999–2006) serotype 3 was most commonly isolated in children older than 24 months (61.2% vs 40.6%;*P *= 0.05) and showed a lower rate of penicillin non-susceptibility (4.0% vs 18%; *P *= 0.003).

**Conclusion:**

*Streptococcus pneumoniae *serotype 3 is an important pathogen in Costa Rican children with otitis media, especially in children older than 24 months of age (*P *= 0.05). Most serotype 3 isolates were susceptible to penicillin, cephalosporins, macrolides and quinolones.

## Background

*Streptococcus pneumoniae (S. pneumoniae) *is one of the most common bacterial pathogens producing invasive and non-invasive disease in healthy and immunocomprised hosts [1]. Invasive pneumococcal disease (IPD) can lead to high morbidity and mortality and according to data from the World Health Organization is the most frequent cause of vaccine preventable mortality particularly in developing countries [2].

Otitis media (OM) represents the most common non-invasive disease caused by *S. pneumoniae *[1]. It has been estimated that the overall incidence of acute otitis media is 1.4 episodes/year/child [3]. Among these children, 20% will develop recurrent otitis media (ROM) and 10% will represent an otitis media therapeutic failure [3]. *S. pneumoniae *is responsible for approximately 38% of all cases of OM worldwide and 30 to 61% of OM episodes in Latin American children [3-5].

Recently a seven valent-pneumococcal conjugated vaccine (PCV-7) containing serotypes 4, 6B, 9V, 14, 18C, 19F and 23F, has been introduced into the National Immunization Programs in different parts of the world [6-9]. This vaccine has shown to be effective in reducing pneumococcal disease burden in countries where it is offered [6-9]. Unfortunately its introduction has being postponed in developing countries where a high number of children are at risk for non-invasive and invasive disease [9].

Following the introduction of the PCV-7 in various regions of the world, an important decline in the number of invasive *S. pneumoniae *disease [8] and a moderate decrease in the number of OM episodes have been reported [10-12]. Kaiser Permante Study group have reported an efficacy of 7% reduction in the number of acute otitis media (AOM) episodes, 22.8% reduction in frequent otitis, and 20% reduction ventilatory tubes placement with an overall 67% vaccine serotype otitis media efficacy [11]. Besides, in Finland a 57% of PCV-7 vaccine efficacy against vaccine type (VT) serotypes was found in AOM episodes and a 39% decrease in ventilator placement was reported [10]. Zhou at al demonstrated a 42.7% and 41.9% reduction in rates of ambulatory visits and antibiotic prescriptions associated to AOM, respectively, four years after universal PCV-7 use in the United States [13].

In spite of the PCV-7 efficacy, a serotype replacement phenomenon by non-vaccine serotypes (NVT) has been described [14,15]. Serotype 3 has been reported as one of the most common middle ear fluid (MEF) replacement isolates [15] and as a common cause of necrotizing pneumonia [16]. Furthermore, other studies have clearly indicated that, when detected in the nasopharynx, serotype 3 is prone to produce OM [17] and a recent study with an eleven-valent vaccine showed that because of the big capsule, antibodies induction against this serotype is difficult [18,19].

This study, describes the clinical, epidemiological and antimicrobial resistance patterns of serotype 3 produced-OM in Costa Rican children and its role in OM.

## Methods

### Study population

The present study was a retrospective analysis of an otitis media data base, which includes demographic, clinical (including vaccination records) and bacteriological information. The study population included children with otitis media who participated in different OM antimicrobial clinical trials between 1992 and 2007 in Costa Rica, in whom a MEF sample for bacterial culture was obtained. All tympanocentesis were done previous to the use of antibiotics. None of the patients received placebo during any of the clinical trials. For the analysis of this study, each bacterial isolated was counted only once.

All the studies were approved by one of the following Institutional Review Boards (IRB) or Independent Ethics Committee (Costa Rican Social Security Institutional Review Board; Universidad de Ciencias Médicas Ethical Committee and CIMA Hospital Ethical Committee. Also, for every participant written informed consent was obtained from the parent or legal representative before inclusion into each study.

Pneumococcal conjugated seven valent vaccine was introduced in year 2004 in a limited sector of Costa Rican children in year 2004 (10% to 20% of children). None of the study participants have received the seven valent-pneumococcal conjugated vaccine.

#### Definitions

The following definitions were used for the analysis of children included in the present analysis: otitis media; children with at least one of the following ear findings: purulent otorrhea for less than 48 hours or at least two otoscopic signs of middle ear effusion (decreased or absent tympanic membrane mobility, yellow or white discoloration of tympanic membrane or opacification of tympanic membrane) and at least one indication of acute inflammation (ear pain, marked redness of tympanic membrane or distinct fullness or bulging of tympanic membrane); Acute otitis media (AOM), signs and symptoms of otitis media for ≤ 72 h; recurrent otitis media (ROM), history of 3 or more episodes of OM in the last 6 months or 4 or more episodes in the last 12 months (including the current episode); and failure otitis media (FOM), persistence of signs and symptoms of OM for 48 hours despite adequate antimicrobial therapy or an episode of OM occurring within 14 days after the last dose of an antimicrobial prescribed for an OM episode.

### Middle ear fluid sampling

Tympanocentesis was performed as previously described [4,5]. In case a participant had a perforated tympanic membrane, removal and cleaning of the ear canal material was done and deep aspiration of the MEF material was attempted. MEF samples were immediately transferred to the local research laboratory in amies medium without charcoal (Copan diagnostics inc., Corona, ca^®^) for processing.

### Microbiology

Inoculation of the middle ear aspirate was performed onto: blood agar, chocolate agar, McConkey agar and mannitol salt agar for incubation at 35°C in a 5% CO_2 _environment with a relative humidity of 80–90% for 18 to 72 h at the local laboratory facilities of Centro de Investigaciones Clínicas, San José, Costa Rica. If no growth was observed after 72 h, the culture was considered sterile. If growth was present on solid media, identification was performed by standard procedures [20]. For the analysis of this study, *S. pneumoniae*, Non-Typable *Haemophilus influenzae (NT H. influenzae), Moraxella Catarrhalis (M. catarrhalis) *and *Streptococcus pyogenes *(*S. pyogenes*) were considered target MEF pathogens, all other isolates were considered as potential contaminants. All Non-Typable *H. influenzae *and *M. catarrhalis *isolates were tested for beta-lactamase production with the use of the cefinase disk.

#### Streptococcus pneumoniae isolates serotyping

Isolates were stored at -70°C at the Centro de Investigaciones Médicas in Costa Rica using Micro Bank vials (Pro-Lab Diagnostics, Austin, TX) until shipped on dry ice or transport medium to the Pediatric Infectious Disease Research Laboratory at the Soroka University Medical Center, Beer-Sheva, Israel where serotyping was performed by the quellung reaction using antisera from Statens Serum Institute of Copenhagen, Denmark [20]. Serotyping was performed only in pneumococcal isolates recovered from the middle ear between years 1999 and 2006. Strains collected before 1999 were not available for analysis and strains from 2007 have not been serotyped yet.

#### Antimicrobial susceptibility

Susceptibility testing of *S. pneumoniae *isolates was performed for penicillin, amoxicillin, cefuroxime, ceftriaxone, azithromycin, and levofloxacin and determined by minimal inhibitory concentration (MIC) by means E-test (PDM Epsilometer, AB Biodisk, Solma, Sweden). Susceptibility testing and interpretation for MIC breakpoints was done following the Clinical and Laboratory Standards Institute (CLSI) guidelines [20]. Susceptibility tests to penicillin were done using the cut-off for oral treatment: MIC value of ≤ 0.06 mg/L were considered susceptible to penicillin (PSSP); isolates with an MIC value between 0.125 and ≤ 1.0 mg/L were considered intermediate to penicillin (PISP) and those with an MIC value ≥ 2.0 mg/L were defined as penicillin-resistant (PRSP).

#### Statistical analysis

The Statistical package EPI INFO (version 6.0) was used to test differences (by the Fisher's exact test, Yates Chi Square Test, as appropriate). A *P *value of < 0.05 was considered significant

## Results

### Study population

Among 1919 children with otitis media, 1208 bacterial pathogens were obtained from the MEF during the study period (Table 1). Acute otitis media (AOM), recurrent otitis media (ROM), failure otitis media (FOM) and FOM/ROM represented 72%, 17.0%, 8.3% and 2.4% of all cases, respectively. None of the study participants have received the seven valent-pneumococcal conjugated vaccine.

**Table 1:** Total population according to different study analysis steps

**STUDY POPULATION**	**YEARS**	**TOTAL**	**PERCENTAGE**
Tympanocentesis performed	1992–2007	1919	
Bacterial pathogens isolated	1992–2007	1208/1919	62.9%
*S. pneumonia *positive cultures	1992–2007	511/1208	42.3%
*S. pneumonia *serotyping	1999–2006	346/511	68.0%

The most common pathogens were *S. pneumoniae*, 511 isolates (42.3%); *NT H. influenzae*, 386 isolates (32%); *M. catarrahalis*, 100 isolates (8.2%); and *S. pyogenes*, 54 isolates (4.47%); other pathogens were isolated in 157 children (13.0%).

Among the 511 patients where *S. pneumoniae *was isolated, information regarding the type of otitis media type was available for 460 children; *S. pneumoniae *was isolated in 347 (75.4%) of AOM children, 77 (16.7%) of ROM children, 28 (6.0%) of FOM children and in 8 children with history of ROM and with a current FOM episode (1.7%).

### Streptococcus pneumoniae characteristics

**Figure 1:**
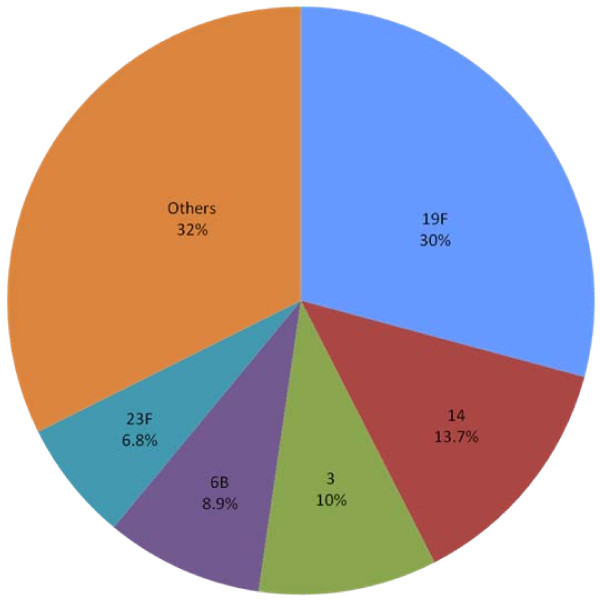
**Serotype distribution among 346 *Streptococcus pneumoniae *isolates from Costa Rican children with otitis media 1999–2006**.

*S. pneumoniae *serotyping was performed in 346/511 (68%) MEF isolates recovered between 1999 and 2006; as explained, in the remaining 32% of the pneumococcal isolates serotyping was not performed. The most common serotypes were 19F (101/30.0%), 14 (46/13.7%), 3 (34/10.1%), 6B (30/8.9%), and 23F (23/6.8%). Other serotypes represented 112 isolates (32%) (Figure 1).

### Serotype 3 Streptococcus pneumoniae characteristics

*S. pneumoniae *serotype 3 was recovered from 34 MEF samples representing 10% of all pneumococcal isolates during the study period; however, this serotype became more prominent during years 2004 and 2005 as compared to the other study years (Table 2). Ninety six percent of *S. pneumoniae *serotype 3 isolates had a mucoid macroscopic appearance

**Table 2:** *Streptococcus pneumoniae *serotype 3 isolates from Costa Rican Children with Otitis Media (1999–2006).

**YEAR**	**ALL *S. pneumonia***	**SEROTYPE 3**	**PERCENTAGE OF TOTAL**
1999	22	1	4.5
2000	13	3	9.6
2001	31	2	15.3
2002	29	3	10.3
2003	65	3	4.6
2004	74	13	17.5
2005	28	5	17.8
2006	84	9	10.7

**TOTAL**	**346**	**34**	**9.82**

When comparing the serotype 3 isolates with the other pneumococcal serotypes (control group), 62% of children with serotype 3 otitis media were older than 24 months whereas in the control group this percentage was 41% (*P *= 0.05). Distribution of serotype 3 per clinical diagnosis was: 26 (76%) AOM children; 1 (3%) FOM children and 7 (20%) ROM children and in 18 patients the disease was bilateral. No statistical difference was found when otitis media clinical diagnosis and laterality presentation were compared between serotype 3 OM episodes to OM episodes by another serotype.

### Antimicrobial susceptibility

Penicillin susceptibility testing was performed in 28/34 serotype 3 isolates. Ninety six percent of serotype 3 isolates (27/28) were penicillin susceptible isolates (PSSP) and one was intermediate; however, only 79% (239/303) of other *S. pneumoniae *serotypes were penicillin susceptible (*P *= 0.003).

All *S. pneumoniae *isolates where fully susceptible to other antibiotics: amoxicillin, cefuroxime, ceftriaxone, faropenem, azithromycin and levofloxacin.

### Clinical Outcome

Per the different study protocols, no statistical difference was found in the clinical and bacteriological outcome at the end of treatment (days 11–14) and at the end of study visit (days 28–32) among patients with OM caused by serotype 3 compared to the clinical and bacteriological outcome observed in patients with OM with other serotypes.

Eighty-nine percent (26/29) of patients from whom *S. pneumoniae *serotype 3 was isolated were cured or improved at the end of treatment visit 82.7% (24/29) remained cured at the end of study visit. Similarly, among children in whom another MEF pneumococcal, the percentage of children cured was 85.5% (186/218) and 79.8% (174/218) remained cured at the end of study visit.

## Discussion

Otitis media represents one of the principal causes for medical consultation and antibiotic prescription in the pediatric population. It has been estimated that in the USA this disease is responsible for approximately 30 million outpatient visits per year, representing an economic impact of more than 2 billion dollars per year [3].

*S. pneumoniae *is the most common pathogen producing OM in children worldwide [3]. To date, > 90 pneumococcal serotypes have been reported [1]. Because the introduction of PCV7 has been associated with a serotype replacement phenomenon in some countries [14,15], it is important to perform regular serotype analysis surveillance among children with OM to predict the potential impact of the vaccine and establish serotype natural dynamics.

This study analyzed the serotype distribution of *S. pneumoniae *recovered from the MEF of Costa Rican children with OM and demonstrated that *S. pneumoniae *serotype 3 was recovered from 34 MEF samples representing 10% of all pneumococcal isolates between 1999 and 2006 with the typical yearly fluctuation characteristics, becoming particularly common among Costa Rican Children during years 2004, 2005 and less common during 2006. These serotype yearly fluctuations are particularly important. However, these results must be interpreted with caution because only 68% of all *S. pneumoniae *isolates were serotyped, serotype 3 representing 6.7% of all neumococcal isolates serotyped, which can introduce a biass to the data analysis. In spite of this, when analyzing serotype 3 frequency among the study years, there was a yearly fluctuation and represent the foundations for a continues surveillance even after the vaccine is introduce in a specific region.

Although in only 63% of the patients a bacterial isolated was recovered, the use of antibiotics as part of the clinical trials didn't influences the results due to the tympanocentesis was performed previous to the antibiotic use.

The introduction of the seven valent conjugated pneumococcal vaccine in the USA has shown to be very effective decreasing the incidence of invasive pneumococcal disease in children. However, new non-vaccine serotypes, such as serotype 3, have emerged as cause of disease [15]. This is particularly important because creating a vaccine that elicits appropriate and effective antibodies against serotype 3 may be difficult because of the large size capsule this serotype exhibits. Even more, a recent trial with an eleven valent vaccine that contained antigens for serotype 3 failed to elicit good antibody levels [18,19]. A 13 valent vaccine with antigens for serotype 3 is under consideration by different regulatory agencies but still is not yet available.

Similar to previous studies, our results also show that sixty percent of children with OM due to *S. pneumoniae *serotype 3 were older than 24 months at diagnosis and 96% of the isolates had a macroscopic mucoid appearance. The presence of this mucoide apperance could be an important virulence factor which could limit the effectiveness of the pneumococcal vaccine for otitis media [21].

Antimicrobial susceptibility results demonstrated that 94% of serotype 3 isolates were PSSP but only 79% of other serotypes were PSSP. There was no susceptibility differences with the other antibiotics tested. This finding suggests that, although serotype 3 has increased as major OM pathogen in Costa Rican children > 24 months of age, common antimicrobial agents such as amoxicillin should be considered the first therapeutic option to treat these patients.

## Conclusion

In conclusion *S. pneumoniae *serotype 3 has increased as otitis media pathogen in Costa Rican children with OM; this should be considered when introducing any future pneumococcal vaccine in the National Vaccines Programs.

## Competing interests

The authors declare that they have no competing interests.

## Authors' contributions

AA participates as sub investigator in some of the clinical trials and performed the data base analysis. CS participates as sub investigator in some of the clinical trials and contributed to the manuscript writing. SG participates as sub investigator in some of the clinical trials and performed the data base analysis. NP performed the serotyping. RD contributed to the manuscript writing. AA participates as principal investigator in all clinical trials and contributed to the manuscript writing. All authors read and approved the final manuscript.

## Pre-publication history

The pre-publication history for this paper can be accessed here:


